# Structure Models of Metal Melts: A Review

**DOI:** 10.3390/ma17235882

**Published:** 2024-11-30

**Authors:** Ailong Jiang, Yujuan Li, Qihua Wu, Yusheng Qin, Shixuan Ma, Yunji Zhang, Xiaohang Lin, Xuelei Tian

**Affiliations:** 1 State Key Laboratory of Engine and Powertrain System, Weichai Power Co., Ltd., Weifang 261001, China; jiangal@weichai.com (A.J.); liyujuan01@weichai.com (Y.L.); wuqh@weichai.com (Q.W.); qinyusheng@weichai.com (Y.Q.); mashixuan@weichai.com (S.M.); zhangyunji@weichai.com (Y.Z.); 2 Key Laboratory for Liquid-Solid Structural Evolution and Processing of Materials, Ministry of Education, School of Materials Science and Engineering, Shandong University, Jinan 250061, China

**Keywords:** physical model, melt structure, short-range ordering, alloy

## Abstract

Nowadays, metallic materials are subject to increasingly high performance requirements, particularly in the context of energy efficiency and environmental sustainability, etc. Researchers typically target properties such as enhanced strength, hardness, and reduced weight, as well as superior physical and chemical characteristics, including electrochemical activity and catalytic efficiency. The structure of metal melts is essential for the design and synthesis of advanced metallic materials. Studies using high-temperature liquid X-ray diffraction (HTXRD) have established a broad consensus that short and medium range ordering exists within metallic melts. However, the high-temperature and liquid conditions during experiments obscure the fundamental physical characteristics, leading to ongoing discussions. Developing simplified models is a typical approach to deal with the complex systems, facilitating a clearer and more direct understanding of the underlying physical images. Here, different physical models of metal melts will be reviewed, starting with transient models, then following with thermodynamic statistical model. The physical image and applications of the models will be carefully discussed.

## 1. Introduction

In modern industrial production, metal products are widely used across various fields such as aerospace, automotive/machinery manufacturing, precision instruments, and rail transportation. They occupy a significant share of the manufacturing market, especially steel and aluminum alloy products. In 2022, the crude steel production of 64 major steel-producing countries worldwide (accounting for approximately 98% of total production) reached 1911 million tons. In 2023, the global output of primary aluminum was 69.7587 million tons, and in the first five months of 2024, production reached 35.84 million tons. In recent years, increasing pressures on the environment, ecology, and resources have imposed higher demands on metal products. These demands mainly focus on the following three aspects: (1) Light weight with higher strength and hardness; for instance, high-speed railways’ increased speed has placed greater requirements on the quality of tracks and train bodies, while the aerospace sector also requires high strength-to-weight ratios for fuselages; (2) Electrode materials with energy storage capabilities: electrode materials such as sodium (Na) and potassium (K) have been deeply investigated recently, as well as lithium (Li) batteries, which have already been widely adopted in the new energy sector [1,2]; (3) Alloys with special properties; this includes alloys with unique characteristics such as electrical, optical, acoustic, magnetic, and thermal properties, for example, high-entropy alloys [3,4,5,6], high-temperature alloys [7,8,9], and amorphous alloys [10,11,12,13,14,15].

The design, research, and development of metal materials are primarily controlled through two aspects: composition design and preparation processes (such as casting, forging, welding, and powder metallurgy). Obviously, not only traditional alloy materials but also emerging novel materials (such as amorphous, quasi-crystalline [16,17,18], and nanocrystalline [19,20,21] materials) undergo a solidification process during their preparation, which involves the transformation of metal from the liquid state to the solid state. In other words, liquid metals serve as the “parent state” for almost all metal products. Therefore, control of the composition and processing of metal material products fundamentally involves altering the liquid structure, properties, and their evolution during solidification. Understanding the liquid structure of alloys and the evolution of their liquid properties is crucial for enhancing the overall performance of metal products. This understanding forms the foundation for developing high-performance novel alloys and is key to such improvements.

Since the 1950s, with the development of high-temperature liquid X-ray diffraction (HTXRD) technology, it has been recognized that metallic melts do not possess a completely “disordered structure” but rather have a short-range ordering distinct from the long-range translational symmetry of crystals. Since then, researchers have attempted to study the microscopic structures of metallic melts through experimental methods. Among these methods, high-temperature liquid X-ray diffraction (HTXRD) is the oldest and most commonly used technique for melt research, as it allows a relatively intuitive observation of atomic structures from reciprocal space (the frequency space that describes the periodic patterns of lattice repetition). HTXRD can provide reliable information on key structural parameters, such as the cluster correlation radius [22,23,24,25,26,27,28,29,30] (which can generally be considered the cluster size), as well as interatomic distances and coordination numbers. Additionally, X-ray absorption spectroscopy and extended X-ray absorption fine structure (XAS and EXAFS) are also commonly employed research techniques [31,32,33,34,35]. These methods obtain information about atomic distances, coordination numbers, and types of nearby atoms by measuring the absorption of X-rays by the material. Other common methods include neutron scattering [36,37,38], among others. With the enhancement of computing power and the development of related simulation techniques, molecular dynamics simulations (MD) [39,40,41] and Monte Carlo simulations (MC) [42,43,44,45,46,47] have also become important tools for studying the structure of melts.

However, the inherent characteristics of metallic melts, such as high temperature and fluidity, pose significant challenges for experimental research and analysis. The intrinsic properties of the melts can easily be obscured by complex experimental phenomena, making it difficult to understand the structural features of the melts on the atomic level through experiments. This has led to a limited understanding of the structures of liquid metals. Therefore, it is necessary to establish reasonable physical models to simplify and describe the structure of metallic melts under ideal conditions. Through mathematical modeling techniques, extensive inductive reasoning, and advancements in experimental technologies, researchers have managed to gain insights into the structure of melts. Note that most research focuses on the structure of molten metals and alloys at temperatures far from the corresponding liquidus temperatures without the occurrence of nucleation processes.

In 1959, J.D. Bernal [48] became the first to attempt to describe liquid structure using graphical modeling techniques, highlighting the validity of statistical description and transient configuration description of liquid structures. This paper will discuss the research progress in statistical description and transient configuration description from the perspective of physical models of alloy melts. Regarding transient configuration description, this paper will focus on widely used models such as the random close-packed hard sphere model and the comprehensive cluster model. Then following with thermodynamic statistical model, particularly the latest statistical description model—the Wulff cluster model. Finally, we will provide an outlook on research regarding alloy melts.

## 2. Transient Models of Alloy Melts

### 2.1. Random Close-Packed Hard Sphere Model

**Figure 1:**
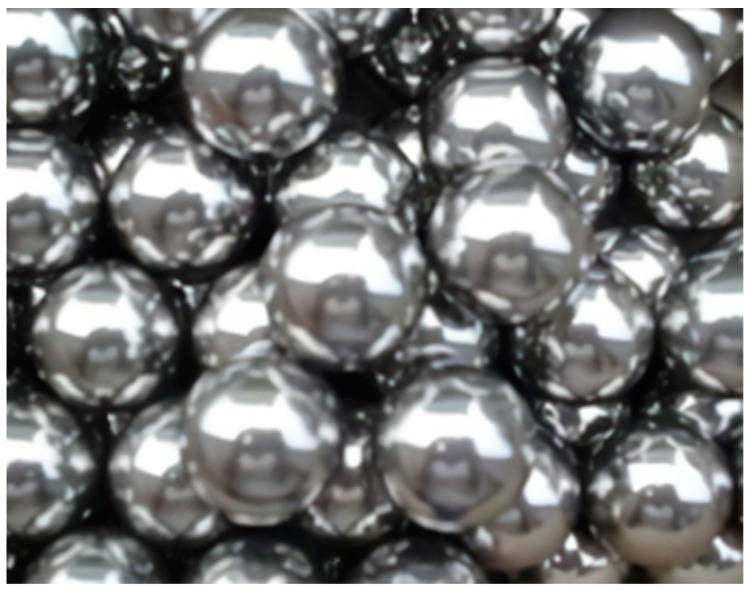
An example of the random close-packed sphere model based on the hard sphere model.

Unlike the long-range ordered structure found in solid crystals, the structure within metallic melts is generally considered to be disordered or only short-range ordered. Therefore, in the early stages of studying melt structures, due to limitations such as experimental conditions, the models used to describe the structural characteristics of melts were primarily based on hard sphere models, which represent atoms as hard spheres to describe the ordered structures within the melt. This approach originated in 1936, when Morell [49] used soft rubber balls to represent the microscopic particles of liquids, thereby obtaining a three-dimensional microscopic image of liquid structure. Building on this, Bernal [48,50] proposed the random close-packed (RCP) model based on the hard sphere model in 1960. This model suggests that the structure of a melt can be compared to the scenario of pouring many rigid ball bearings into a container with an irregular surface and shaking it until they can no longer be compressed, resulting in the formation of a certain structure. The closest random packing occupies approximately 64% of the total volume, as illustrated in Figure 1.

**Figure 2:**
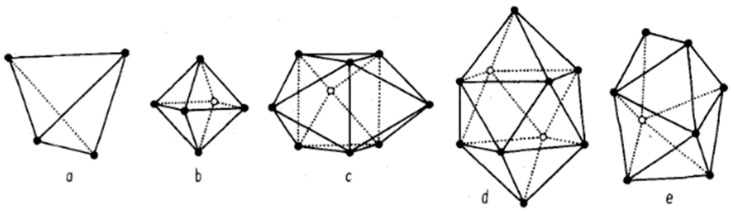
Polyhedral structures corresponding to five hard sphere models given by J.D. Bernal [53]: (**a**) Archimedean antiprism, (**b**) triangular prism, (**c**) quadrangular dodecahedron, (**d**) tetrahedron, and (**e**) octahedron. This figure has been reprinted with permission from the corresponding journal.

Based on the close-packing method mentioned above, J.D. Bernal et al. [51,52,53,54] established five common polyhedral structures, as shown in Figure 2, to describe the hard sphere RCP model and calculated the radial distribution function for this type of model. Subsequently, attempts were made to apply the hard sphere RCP model in various microscopic systems, significantly simplifying the research difficulty. This random and disordered topological model, which determines the coordinates of sphere centers easily, is convenient for mathematical modeling and calculations. Furthermore, the computational results based on this model were found to be close to experimental results, and at that time, it was regarded as the standard model for describing metallic melts and amorphous materials. However, the hard sphere RCP model is based on the assumption of identical hard sphere radii and ignores the interactions between atoms that exist in real metallic melt environments. Therefore, this model can give only a simple description of the arrangement of atoms and is not applicable to complex metallic melts, especially multi-component alloy melts. Nevertheless, this does not detract from the model’s pioneering role in the conceptualization of complex melt structures. Due to its ease of computation and application, along with an experimental error typically not exceeding 15% [55], it was widely promoted for a period and popularized among researchers the convenience of the pair distribution function as a mathematical tool.

### 2.2. Microcrystalline Model

With the development of metallic melt theory, it has gradually been recognized that metallic melts still retain some properties of their solid crystalline structure. Subsequently, several models based on crystalline structures were proposed to describe the structures of metallic melts.

(1) Microcrystalline Model: This model posits that metallic melts are composed of many small crystals, which are connected by lattice plane defects. By combining these small crystals, a complete lattice structure can be formed [56,57].

This model is suitable for describing the short-range ordered structures within the amorphous system. J. Eileen et al. [56] investigate the recrystallization of glasses synthesized from Illinois coal fly ash. They found that the glasses could not be recrystallized to more than approximately 23 vol%, which could be explained by the limited amount of TiO_2_.

(2) Vacancy Model: This model suggests that as temperature increases, solid metals gradually melt into liquid metals, during which their lattice experiences the formation of a considerable number of vacancies due to the increase in energy. The number of vacancies within the lattice will continue to increase with rising temperature, until the complete lattice is “torn apart”, thereby breaking the long-range ordered characteristics of the solid lattice [58,59].

The increase in vacancies corresponds to a macroscopic characteristic of the melt, which is a decrease in viscosity. F.H. Stillinger et al. [58] studied the system softening phenomena caused by the process of excitation of defects upon repetition. They constructed a simple theory that the solid–liquid phase transition is caused by defect softening. The melting point could be correctly calculated by the theory.

(3) Dislocation Model: Similar to the vacancy model, during the melting process of solid metals, a large number of dislocations are generated, causing the original long-range ordered lattice to distort along the direction of the dislocations and break into fragmented lattices. The emergence of this high concentration of dislocations facilitates the transition of metals from a solid state to a liquid state [60,61].

Microcrystalline models focus on the solid–liquid phase transition. However, due to the significant differences between the actual melt structure and the model description, the model can hardly describe the structure of metal melts.

### 2.3. Cluster Model

Currently, the widely accepted model for describing the structure of metallic melts is the melt cluster model. This model posits that the structure of metallic melts consists of short-range ordered atomic clusters and a large number of free atoms. From a dynamical perspective, there are continuous interactions between these clusters and atoms; some clusters may disappear while new clusters form, leading to a dynamic balance. From a thermodynamic statistical perspective, the numbers of clusters and free atoms maintain a relatively balanced state, with both the size and the number of clusters varying with temperature.

In 2004, D.B. Miracle [62] proposed the method of Face-Centered Cubic (FCC) closest packing to describe the microstructure of metallic glasses. He reproduced the experimental pair distribution function results in the range of 7 Å to 10 Å with high accuracy. He introduced the concept of solvent atom sharing, suggesting that the microstructure of the melt is composed of free solvent atoms and short-range ordered clusters that occupy space in the closest packing configuration. Clusters are characterized by their small size and diverse orientations, while the free solvent atoms fill the remaining space. Although the use of the FCC closest packing method to explain all melts and metallic glasses still faces issues similar to those of the random packing model (i.e., it ignores the uniqueness of molecules), the model’s description of clusters and free atoms remains widely accepted in the academic community and has become one of the fundamental assumptions of the current cluster model, with many related studies still ongoing.

**Figure 3:**
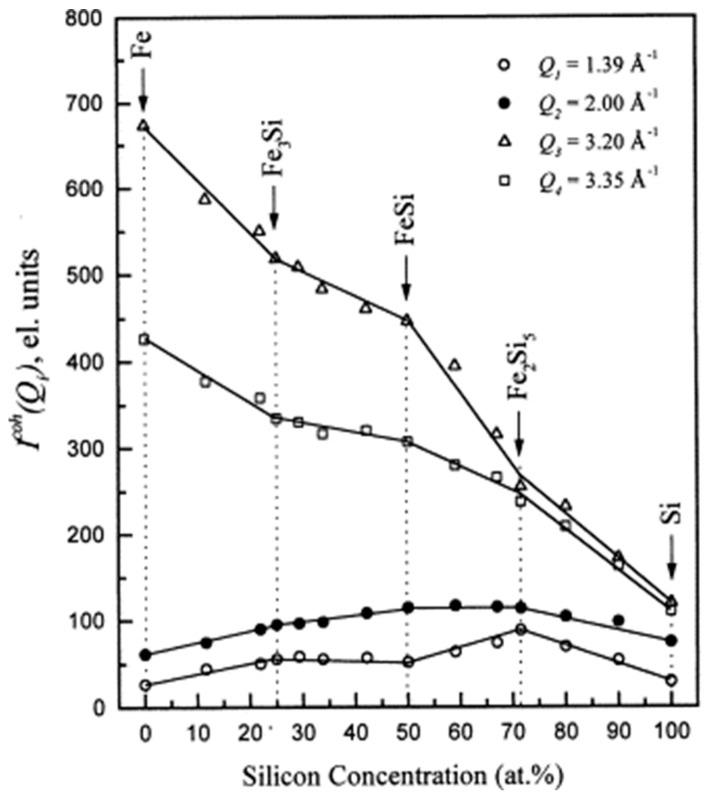
The scattering intensity at a constant value of Q as a function of concentration [63]. This figure has been reprinted with permission from the corresponding journal.

Il’inskii et al. [63] proposed a microscopic inhomogeneous cluster structural model to describe the short-range ordered structure of melts through the study of the structure of Fe-Si melts. They suggested that the non-uniform composition ratios in alloy melts inevitably lead to differences in the shape and arrangement of these short-range ordered cluster structures. They proposed dividing the entire composition range of Fe-Si alloys into several intervals, with each interval exhibiting different types of cluster structures. These types are composed of fixed composition-dependent clusters (Fe clusters, Fe_3_Si clusters, FeSi clusters, Fe_2_Si_5_ clusters, and pure Si clusters, shown in Figure 3). Within the intermediate composition range, the volume fraction of two types of clusters changes with composition, while the atomic arrangement and composition within the clusters remain unchanged. Experimental results from high-temperature X-ray diffraction can validate the accuracy of this cluster description method. If *a* certain alloy exists with two structural clusters, *a* and *b*, then its diffraction intensity can be described as follows:

(1) $$I\left(Q\right)=K^{a}I^{a}\left(Q\right)+K^{b}I^{b}\left(Q\right)$$

(2) $$K^{a}=\frac{c_{1}-c_{1}^{b}}{c_{1}^{a}-c_{1}^{b}},\;K^{b}=\frac{c_{1}^{a}-c_{1}}{c_{1}^{a}-c_{1}^{b}}$$

In the above equation, *I^a^*(*Q*), *I^b^*(*Q*), and *I*(*Q*) represent the diffraction intensities of components a and b, as well as the corresponding diffraction intensity of the binary alloy melt. $c_{1}^{a}$ and $c_{1}^{b}$ represent the concentrations of the first type of atom in the two compositions, while $c_{2}^{a}$ and $c_{2}^{b}$ represent the concentrations of the second type of atom in the two compositions. This model can describe various binary alloy systems such as eutectic, homogeneous, and intermetallic compounds, for example, the Fe-Al, Ag-Sn, and Cu-Sn systems [55,64,65]. Although this method accurately describes the results of X-ray diffraction experiments for binary melts, it unfortunately does not identify the specific cluster model structure at the atomic level.

**Figure 4:**
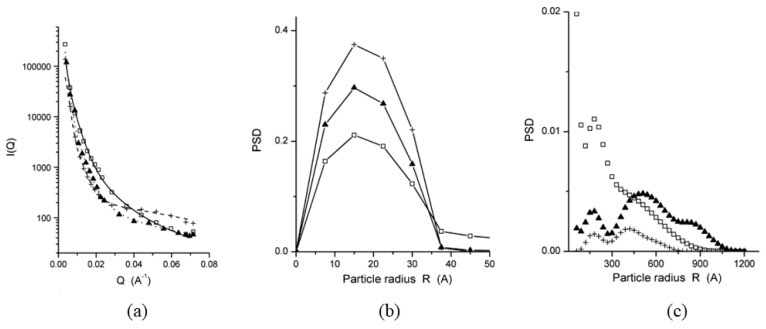
(**a**) Experimental SANS patterns for Al87.8Si12.2 alloy. Volume-weighted particle size distributions of (**b**) small clusters and (**c**) large clusters. The data at 973 K (heating), 973 K (cooling), and 1473 K are represented by open squares, crosses, and filled triangles, respectively [69]. This figure has been reprinted with permission from the corresponding journal.

So far, there have been several studies on the short- to medium-range ordered structural characteristics of binary eutectics and intermetallic compounds, particularly in aluminum-based alloys. W.M. Wang [66] used high-temperature liquid X-ray diffraction (XRD) to investigate the Si atom cluster structure in Al-Si alloy melts. He found that within the sub-eutectic range, the atomic density increased with the Si content, while the coordination number showed an anomalous decrease, reaching a maximum atomic density at the eutectic point. By analyzing the radial distribution function, he confirmed the existence of Si clusters in the eutectic and hypereutectic melts, with the number of clusters initially decreasing and then increasing as the temperature rose [67,68]. U. Dahlborg et al. [69] used neutron diffraction to study Al-Si alloys and discovered an inhomogeneous distribution in the melt above a certain temperature range of the liquidus line. The volume-weighted particle size distributions from experimental SANS patterns directly provide evidence for the existence of cluster structures within the melt (shown in Figure 4). U. Dahlborg et al. [70] and M. Calvo-Dahlborg et al. [71] conducted similar experiments, examining how the number and size of clusters in the melt change with temperature and composition above the liquidus temperature. They provided a unique description: there exists a temperature range above the liquidus line such that at the lower limit of this range, the melt begins to dissolve, and at the upper limit, the melt becomes completely homogeneous.

Regarding the research findings on medium-range ordered structures, X.F. Bian et al. [72,73,74] explored the medium-range ordered structure and its evolution in Al-Fe alloy melts through high-temperature liquid X-ray diffraction. They compared the X-ray diffraction patterns of the Al-Fe alloy melt with the solid Al_5_Fe_2_ intermetallic compound, finding that the structure factor curve of the Al-1%Fe alloy, under low superheating conditions, displayed a pre-peak that indicated the retention of the structure of solid Al_5_Fe_2_ in the liquid metal. This structure disappeared when the superheating reached a certain level, resulting in the corresponding pre-peak on the structure factor curve vanishing as well. J.Y. Qin et al. [75] studied the structural characteristics of Al-Fe alloy melts using the same experimental approach and identified the trend of pre-peak parameters on the structure factor as a function of composition. They used the atomic cluster structural model in the melt to explain the relationship between the pre-peak positions and the composition, reporting, in summary, that there is a close relationship between the structure of Al-Fe alloy melts and the Al-Fe alloy phase diagram.

**Figure 5:**
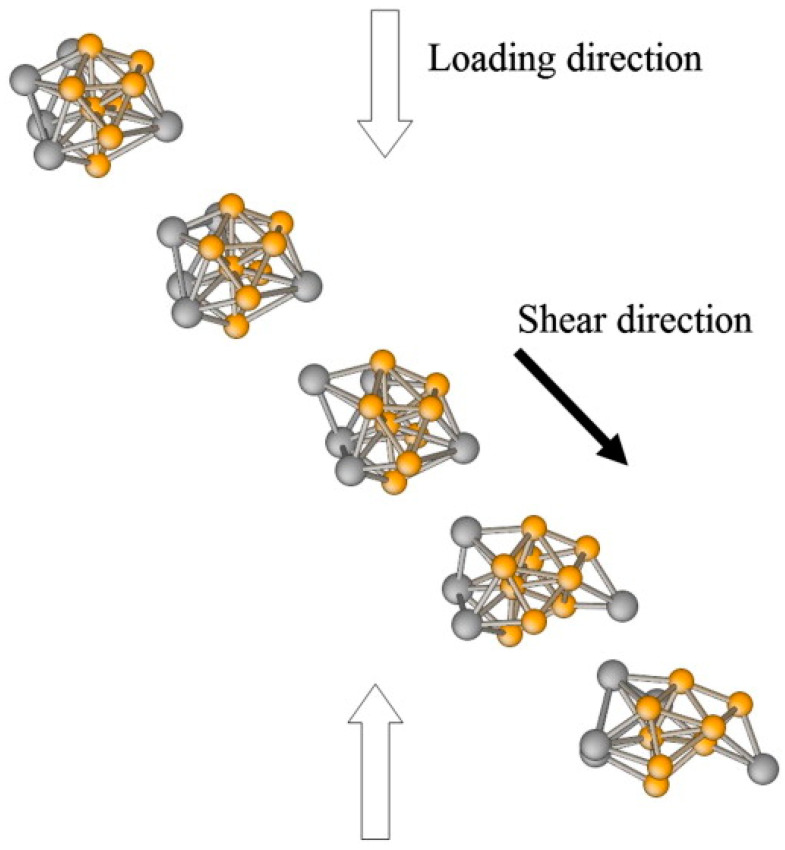
The structural change in a full icosahedral cluster upon joining the localized deformation band. The shear/slip direction (black arrow) and loading axis (open arrows) are represented in the figure. Zr and Cu atoms are colored in gray and yellow, respectively [77]. This figure has been reprinted with permission from the corresponding journal.

After the heterogeneity of medium- and short-range order in alloy melts became a consensus, attempts were made to extract their characteristic structures. H.W. Sheng et al. [76] used synchrotron X-ray experiments and X-ray absorption fine structure (XAFS) experiments to investigate the structural data of Ni_80_P_20_ metallic glass. They reconstructed transient structures matching the experimental results through the reverse Monte Carlo method and then performed Voronoi tessellation on the results, obtaining several representative clusters. They argue in the paper that this method essentially extracts the characteristic structures of metallic glass. However, since the reverse Monte Carlo method treats solute atoms as spheres, many of the structures obtained bear a striking resemblance to Bernal polyhedra. This fails to adequately reflect the influence of atomic uniqueness on medium-range and short-range order. A.J. Cao et al. [77] used molecular dynamics simulations to study the shear deformation of Cu-Zr metallic glass. From their simulation results, they observed a relationship between the degree of deformation of the icosahedron and the shear stage and mode. They noted that the plastic deformation of metallic glass often begins with significant disordering of the regular icosahedron, suggesting that this finding could serve as key evidence for the structure–property relationship of metallic glass (shown in Figure 5). However, this study did not consider the relationship between free atoms and clusters, as it locked the atomic numbering of the atoms composing the regular icosahedron without discussing whether such atoms could be substituted during deformation (i.e., if the atoms could reassemble into a nearby distribution after the original icosahedron disintegrated). Moreover, both Sheng’s and Cao’s theoretical studies face a crucial question when applied to metallic melts: can we use transient modeling as an initial condition and obtain the properties of the melt system in thermodynamic equilibrium through statistical simulation, especially without considering the high free path and high-temperature vibrations of the melt atoms?

**Figure 6:**
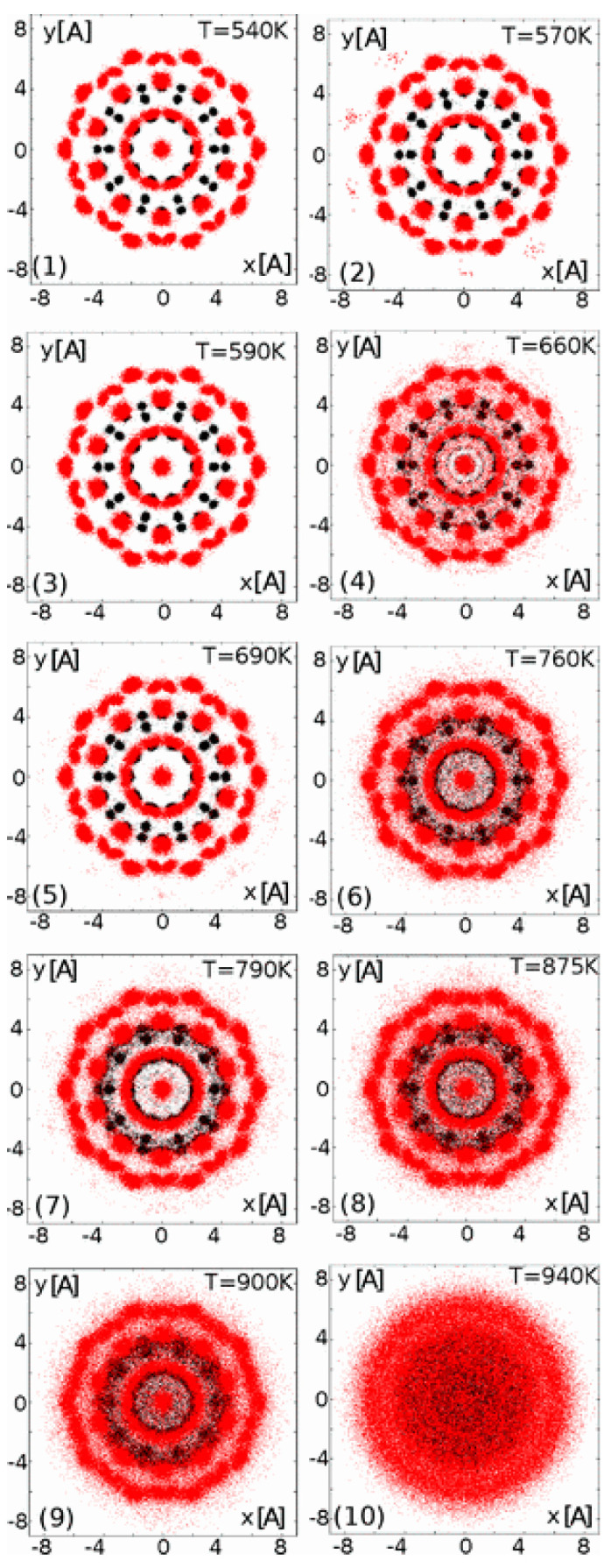
The average topology of Ag_72_Ni_55_ from 5000 snapshots at different temperatures (from 540 K (subfigure (**1**)) to 940 K (subfigure (**10**)) [78]. the black points and gray (red) points correspond to Ni and Ag atoms. This figure has been reprinted with permission from the corresponding journal. (**1**–**10**).

In the studies focusing on the melt itself rather than extending the modeling theories of amorphous structures, researchers tend to extract the characteristic structures that may be contained within the melt instead of comprehensively modeling its transient structure. Z. Kuntová et al. [78] utilized molecular dynamics simulations and a many-body tight-binding potential to investigate the melting enthalpy of icosahedron clusters near magic sizes. By reproducing the characteristic structures and simulating them, they calculated thermal property parameters and partially explained some quantitative differences in melting behavior as well as certain heat capacity anomalies. This signifies that studying characteristic structures rather than modeling the entire melt can be a viable approach for melt research (shown in Figure 6).

Since 2010, the methodology for modeling transient melts has become relatively self-consistent, alongside limitations due to experimental conditions. Consequently, studies on melt structures have primarily concentrated on the effects of the presence patterns of specific clusters on physical properties within particular systems, with many studies often employing molecular dynamics simulation as the research method [79,80,81,82,83,84,85].

However, as research has progressed and expanded, some researchers have recognized that the melt modeling approach based on Bernal polyhedra and icosahedral clusters (hereafter referred to as the quasi-crystalline cluster model) still has its uncertainties. Firstly, although differing from the completely random packing described by Bernal, the quasi-crystalline cluster model still fails to clearly distinguish between molecular types and atomic species; they are treated as hard solid spheres in calculations. This leads to a mismatch in the interpretation of the melt structure and physical properties, where the quasi-crystalline model assumes that the icosahedra and Bernal polyhedra are common to melts, particularly metallic melts. However, explaining specific thermal property parameters for particular systems often necessitates introducing more specific characteristic structures, even if these structures do not perfectly resemble “icosahedra”. While these proposed characteristic structures effectively explain changes in thermal property parameters for melts or amorphous materials, their phenomenological nature makes them difficult to generalize or use for predicting conditions in other systems. Furthermore, the icosahedral polygons proposed by the quasi-crystalline cluster model are often challenging to reproduce even in molecular dynamics calculations. At this point, researchers usually fit the data using deformed icosahedra. However, the issue is that it is relatively easy to find and match a deformed icosahedron from a large number of semi-randomly distributed vertices, provided the coordination density is within an appropriate range (around 12).

## 3. Thermodynamic Statistical Model

Previous research has gradually revealed some limitations of transient structural models. However, studies focusing on characteristic structures are often questioned due to the difficulty of experimentally observing corresponding atomic structures, which is largely attributed to the high free path of melt atoms and their vigorous thermal motion. To address this, X. Lin et al. [86] from Shandong University established the Wulff cluster model, introducing the concept of an equivalent system for structural equivalence under thermodynamic equilibrium.

The Wulff cluster model describes the medium- and short-range structures of melts at thermodynamic equilibrium by considering the most probable structure of alloy melts as an equivalent system. This model posits that the most probable structure of alloy melts should conform to the Wulff shape, while the internal structure should possess symmetry characteristics consistent with crystals. The results of HTXRD experiments of the same melt system are highly consistent, as proved by repeating the experiment several times at each temperature. It obviously proves that the melt systems are in thermodynamic equilibrium. In this case, the shape of short-range ordering with lowest total surface Gibbs free energy can be directly derived from the interface energies by the so-called Wulff construction [87]. Generally speaking, the point group symmetry of the crystals is determined by the distributions of the valence electrons. The properties of electron gas (obeying the Fermi–Dirac distribution) will not change significantly until the Fermi degeneracy temperature. Compared with the Fermi degeneracy temperature (usually higher than 10^4^ K), 0 K and the temperature of metal melts (usually 10^2^ K–10^3^ K) are so low that the trend of point group symmetry hardly changes between 0 K (crystal) and melts (short-range ordering).

X. Lin et al. [86,88,89,90,91] validated the rationality of the Wulff cluster model across various systems by comparing simulated XRD of the Wulff cluster structures derived from the Laue equations (hereafter referred to as simulated XRD) with HTXRD experimental results (hereafter referred to as HTXRD). They studied the medium- and short-range structures of melts in pure metals, binary homogeneous alloys, eutectic alloys, and binary intermetallic compounds.

**Figure 7:**
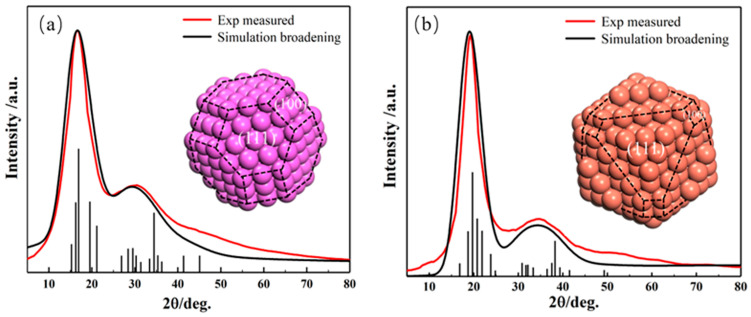
XRD comparison of simulated (black lines) and experimental (red lines) results of the melts of (**a**) Al (**b**) Cu. The corresponding atomic structure is represented in the figure [88]. This figure has been reprinted with permission from the corresponding journal.

In single-phase pure metal systems, the Wulff cluster model can be applied directly without any approximations. According to the HTXRD experimental results, the simulated XRD curves from present models match the experimental results quite well regarding not only the position and width of the peaks but also the relative intensity of the first and second peaks (shown in Figure 7). The Wulff cluster model effectively provides a structural equivalence for metal melt systems [88].

**Figure 8:**
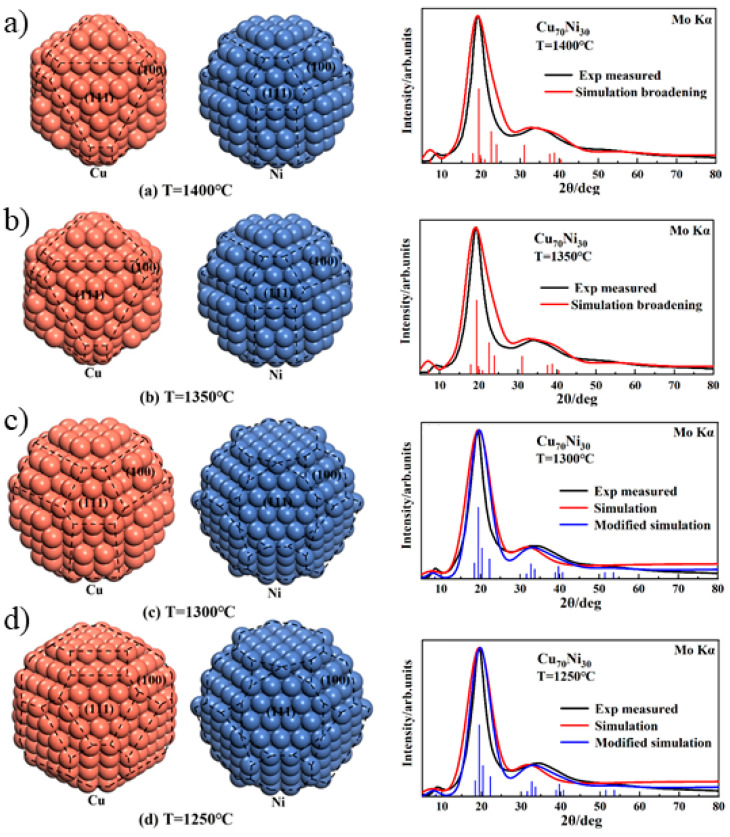
The atomic structure of Wulff clusters in Cu-Ni melts and a comparison of experimental XRD and simulation XRD results at (**a**) 1400 °C, (**b**) 1350 °C, (**c**) 1300 °C, and (**d**) 1250 °C. The black, red, and blue curves represent the experimental, simulated, and modified simulated XRD patterns, respectively [86]. This figure has been reprinted with permission from the corresponding journal.

In the binary homogeneous system, an additional problem arises: whether to consider the presence of different atoms within the same cluster or whether to establish multiple Wulff clusters for different alloy compositions. The modeling assumption here is that since the two components of binary homogeneous alloys are infinitely miscible, their correlation functions can be considered zero, meaning they can statistically be treated as independent. Therefore, in the modeling process, only pure metal clusters are taken into account, and a linear combination is applied based on the alloy composition. This hypothesis has been successfully validated through HTXRD experiments on the Cu-Ni and Au-Ag equiaxed alloy systems, showing excellent agreement with experimental results (shown in Figure 8) [86].

**Figure 9:**
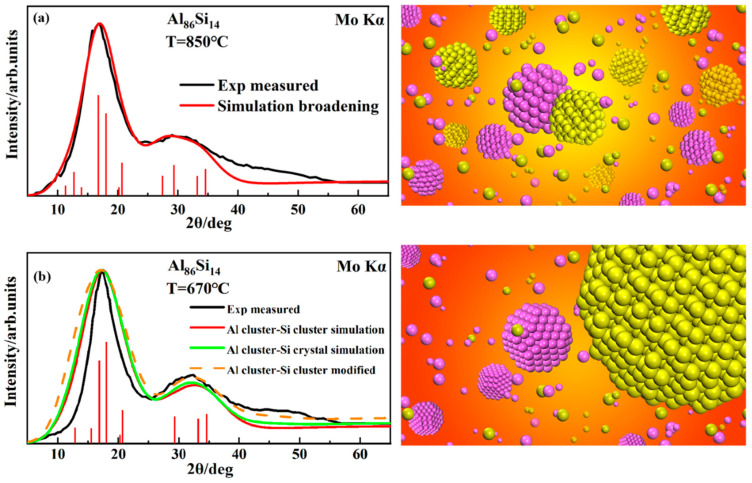
Experimental (black curves) and simulated (red curves) XRD patterns of an Al-Si melt obtained at (**a**) 850 °C and (**b**) 670 °C. The blue curves and the orange dotted line represent the modified simulated results. The figures on the right side illustrate the melt structures at the corresponding temperatures [89]. Al and Si atoms are colored in purple and yellow, respectively. This figure has been reprinted with permission from the corresponding journal.

For eutectic systems, the approach to multi-component clusters remains the same as in the binary homogeneous case. Despite the methodological consistency, the core modeling philosophy differs. In binary homogeneous alloys, the similarity in component properties leads to an effect on structure that approximates linear interpolation, thus allowing them to be treated as independent. In contrast, eutectic alloys exhibit significant differences in component properties, resulting in a low degree of miscibility that can be ignored. Hence, their mixed effects are negligible, allowing them to still be treated as mutually independent components. This modeling philosophy has also been validated through HTXRD for the Ag-Cu and Al-Si systems. Additionally, in the Al-Si eutectic system, another phenomenon was observed: within the temperature range near the liquidus, the simulated XRD results showed a noticeable distortion compared to the HTXRD experimental results. This is hypothesized to arise from the formation of ordered structures with sizes much larger than the X-ray wavelength (λ_Moα_ = 0.7093 Å), which diminishes the size broadening effect and enhances periodicity. By linearly combining the XRD data from a complete block of Si, the distortion was significantly corrected (shown in Figure 9). This indicates that the ordered degree of more stable components near the liquidus line tends to increase, a phenomenon likely related to the nucleation process [89].

**Figure 10:**
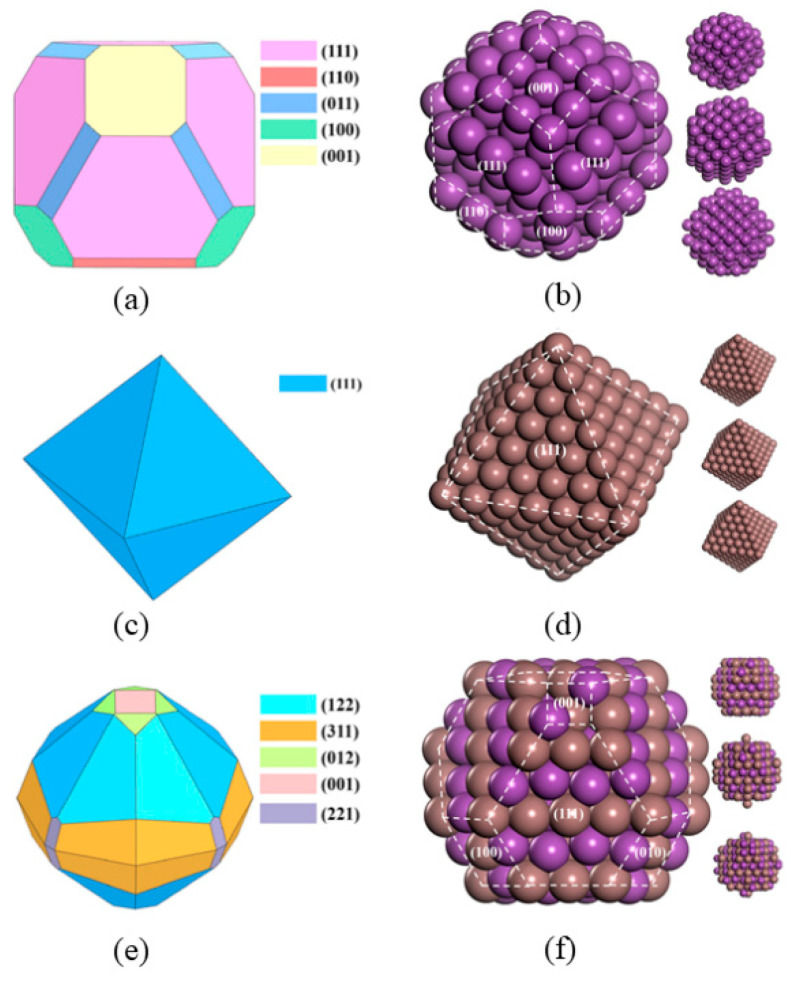
Wulff shapes and atomic structures of possible short-range ordering in an In_50_Bi_50_ melt at different temperatures (180 °C, 160 °C, 140 °C, and 120 °C), including Bi (**a**,**b**), In (**c**,**d**), and InBi (**e**,**f**), respectively [90]. In and Bi atoms are colored in purple and brown, respectively. This figure has been reprinted with permission from the corresponding journal.

**Figure 11:**
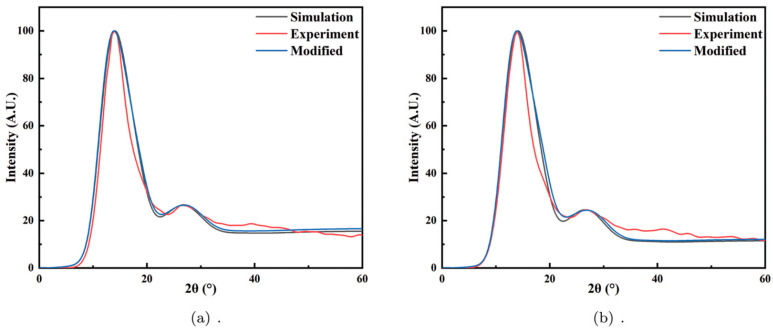
Nucleation modification near liquidus at (**a**) 140 °C and (**b**) 120 °C [90]. This figure has been reprinted with permission from the corresponding journal.

In the ordinary binary alloy systems containing intermetallic compounds, the melt structures of In-Bi and Al-Ti have been studied in depth [90,91]. The presence of intermetallic compounds significantly increases the complexity, as it leads to a greater variety of possible equivalent structures. Taking the In-Bi alloys as an example, with the In_50_Bi_50_ alloy as the research subject, all potential equivalent structures were carefully considered (see Figure 10). By comparing simulated XRD with high-temperature liquid XRD, it was ultimately determined that there existed only InBi clusters and Bi clusters in the melt. The simulated and experimental results matched well, and the nucleation process of the higher-melting-point Bi crystal was observed near the liquidus line (see Figure 11).

**Figure 12:**
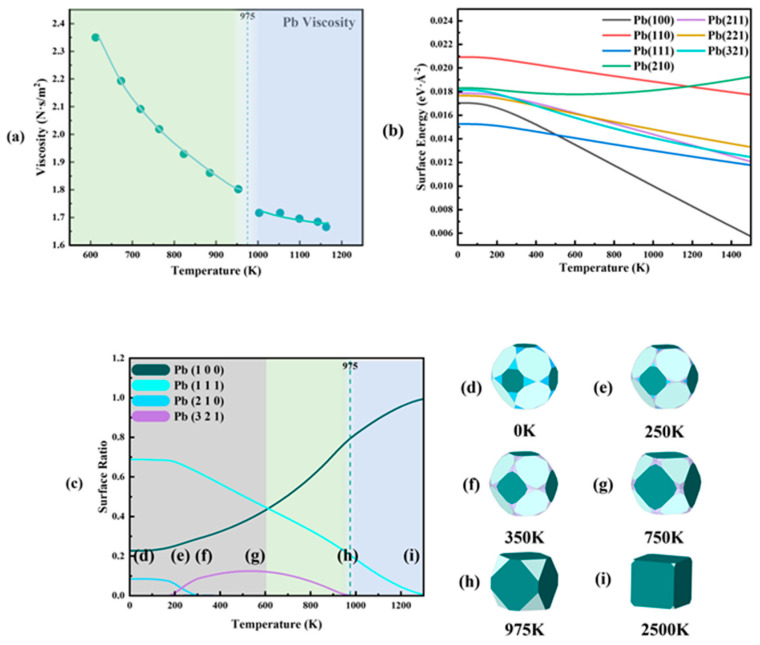
Mechanism of non-Arrhenius viscosity drops of Pb [92]. (**a**) Viscosity–temperature curve of Pb melts, (**b**) Pb surface free energy as a function of temperature, and (**c**) surface ratio of the Pb Wulff shape as a function of temperature. Corresponding Wulff shapes are demonstrated at (**d**) 0 K, (**e**) 250 K, (**f**) 350 K, (**g**) 750 K, (**h**) 975 K, and (**i**) 2500 K. This figure has been reprinted with permission from the corresponding journal.

So far, the Wulff cluster model has been validated in dozens of different alloy compositions and temperature systems. Using this model, we can isolate the ordered portions of the melt and replace them with equivalent structures to study the physical processes related to the melt structure. For instance, this theory allows a reasonable and accurate explanation of the non-Arrhenius transition temperature and type of transition in the viscosity of alloys. Taking Pb and Al as examples, as the temperature increases, the Al(100) plane, with the lowest adsorption energy in the equivalent structure of Al, disappears at 1075 K, which corresponds to a jump in viscosity relative to the predicted values from the Arrhenius equation. Conversely, in the Pb system, the Pb(321) plane, with the highest adsorption energy, disappears at 975 K, corresponding to a sudden drop in viscosity compared to the predicted values (shown in Figure 12). When a plane in the Wulff cluster model of the melt structure disappears or appears, the properties of that plane will determine the type of non-Arrhenius transition phenomenon in viscosity: the disappearance of the Al(100) plane, with the lowest adsorption energy, causes a non-Arrhenius viscosity jump, while the disappearance of the Pb(321) plane with the highest adsorption energy leads to a non-Arrhenius viscosity drop [92].

## 4. Outlook

The Wulff cluster model has achieved considerable success in describing ordered structures and has enabled deeper investigations into the physical processes associated with the ordered portions in the liquid phase. The model may subsequently develop to address the fields of amorphous materials, nucleation, and growth, as well as liquid–liquid phase transitions. However, this model struggles to cope with the physical processes related to the completely disordered portions, such as heat transfer, electrical conduction, and so on. Future research should focus on developing various physical models to tackle the complexity of melt systems. It is essential to organically combine transient models with thermodynamic statistical models to form a comprehensive theoretical framework for melt structure, capable of more fully describing the microstructure and physical properties of melts. The physical models of melt structure will play an increasingly important role in understanding and predicting material behavior, particularly in the field of material design based on product demand in industrial production, thereby providing the theoretical foundation for future material design and engineering applications.
